# Supplementary material for: Chd7 is indispensable for mammalian brain development through activation of a neuronal differentiation programme
Source: Nat Commun. 2017 Mar 20;8:14758. doi: 10.1038/ncomms14758 (PMC5364396; doi:10.1038/ncomms14758)
Supplement: Supplementary Information — Supplementary Figures and Supplementary Tables. [file ncomms14758-s1.pdf]

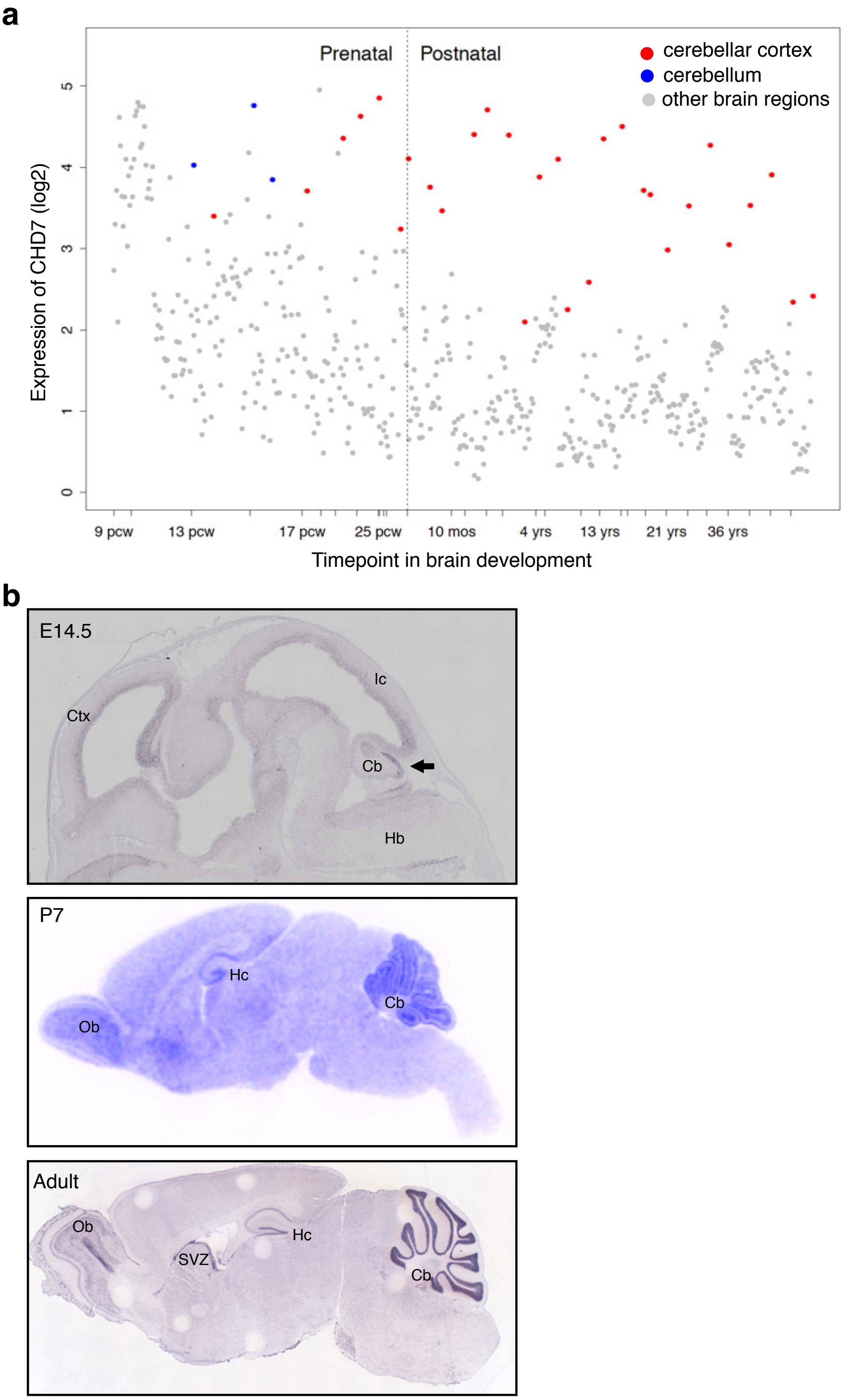

Supplementary Figure 1: Expression of Chd7 in the cerebellum.

(a) The expression of CHD7 is enriched in the cerebellum during human brain development. Data was retrieved from the Allen Brain Institute.

(b) RNA in situ data show the expression of Chd7 in mouse brains at E14.5, P7 and adult. The arrow in the top panel shows the EGL of the cerebellum. The data were retrieved from the following databases: Genepaint (http://www.genepaint.org) for E14.5, Brain Transcriptome database (http://www.cdtdb.neuroinf.jp/CDT/Top.jsp) for P7 and ALLEN Brain Atlas (http://www.brain-map.org) for adult. Ctx, Cerebral cortex. Cb, Cerebellum. Ic, Inferior coliculus. Hb, Hindbrain. Ob, Olfactory bulb. Hc, Hippocampus. SVZ, subventricular zone.

a

Summary of progenies from mating of
Nestin-Cre::Chd7 f/+ x *Chd7 f/f*

	Nestin-Cre:: Chd7 f/f	*Nestin-Cre:: Chd7 f/+*	*Chd7 f/f Chd7 f/+*	Number of litters
Live born	4	23	44	12
E16.5-P0	0	8	10	4
E13.5-E15.5	9	15	16	6

b

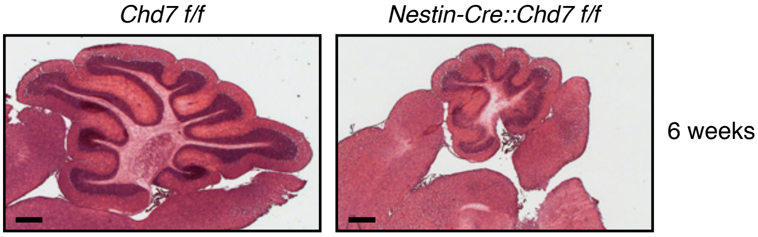

Supplementary Figure 2: Phenotype of *Nestin-Cre::Chd7 f/f* mice.
(a) A summary of genotyping results of progenies obtained from mating of [*Nestin-Cre::*$Chd7^{f/+}$] with [$Chd7^{f/f}$] mice. Note that the number of [*Nestin-Cre::*$Chd7^{f/f}$] mice older than E16.5 is clearly reduced, compared to theoretical ratio calculated with the Mendel's law.
(b) Hematoxylin & Eosin (H&E) staining of [$Chd7^{f/f}$] and [*Nestin-Cre::*$Chd7^{f/f}$] 6-weeks old cerebella. Scale bar, 400 mm.

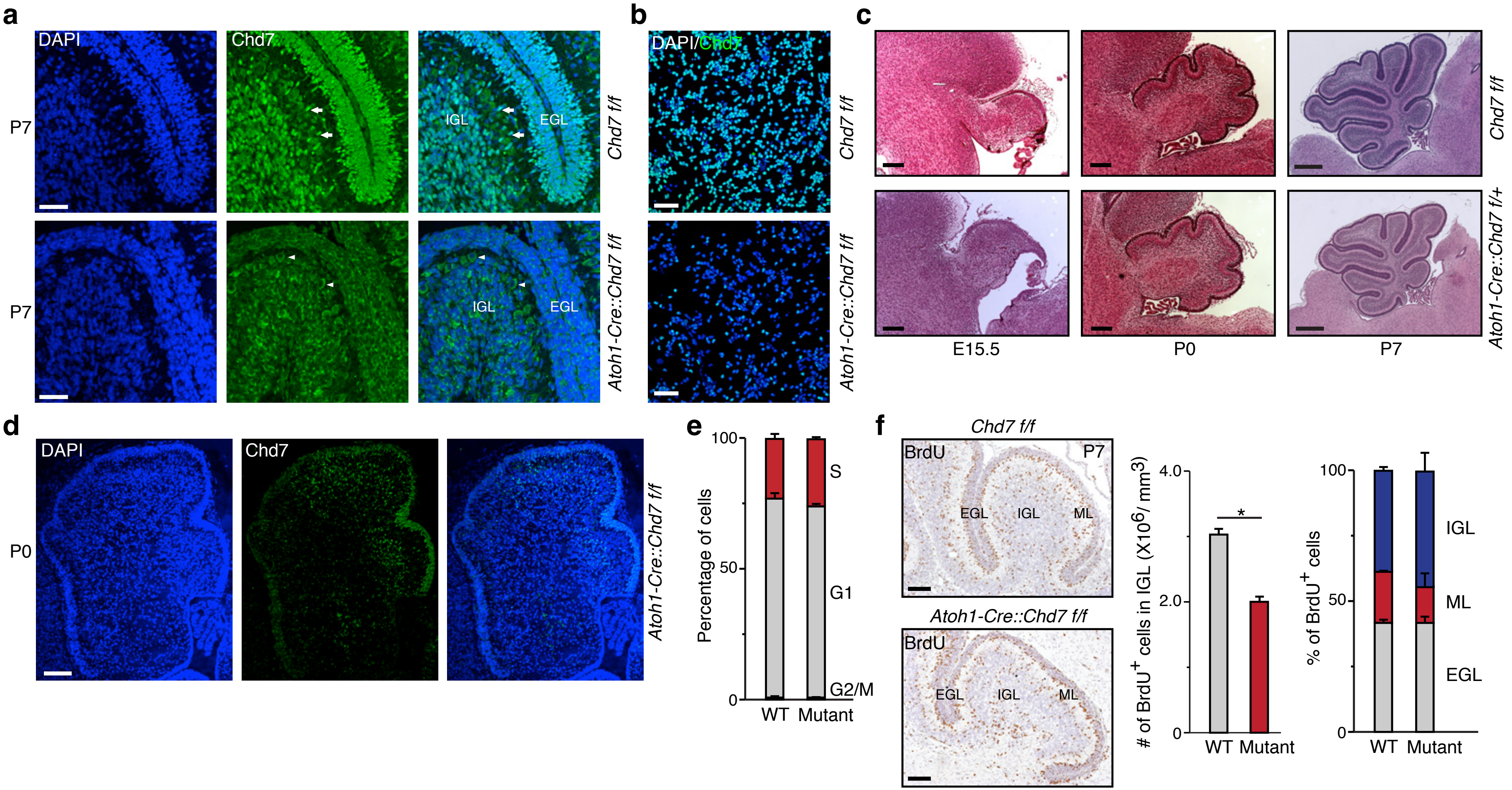

Supplementary Figure 3: Genetic ablation of *Chd7* in cerebellum.

(a) Immunostaining of Chd7 in P7 cerebella of Chd7 WT [*Chd7$^{f/f}$*] and homozygous mutant [*Atoh1-Cre::Chd7$^{f/f}$*]. DNA was stained with DAPI. Note that nuclear staining of the Chd7 antibody is detected in *Chd7* WT granule cells (shown by arrows, top panels), whereas at long exposure time only non-nuclear background staining (shown by arrowheads, low panels) is observed in granule cells of [*Atoh1-Cre::Chd7$^{f/f}$*] mouse. Scale bars, 50 mm. EGL: external granule layer. IGL: internal granule layer.

(b) Immunostaining of Chd7 (in green) in isolated WT [*Chd7$^{f/f}$*] and mutant [*Atoh1-Cre::Chd7$^{f/f}$*] cerebellar granule progenitors (GNPs) cultured without Shh for 48 hours. Note that most of mutant cells lost Chd7 staining. Scale bars, 50 mm.

(c) H&E staining of Chd7 WT [*Chd7$^{f/f}$*] and Chd7 heterozygous mutant [*Atoh1-Cre::Chd7$^{f/+}$*] cerebella at E15.5, P0 and P7. Scale bars, 200 mm.

(d) Immunostaining of Chd7 in P0 [*Atoh1-Cre::Chd7$^{f/f}$*] cerebellum. Note that the Atoh1-Cre-mediated Chd7 deletion is less efficient at the posterior lobes (right side) compared to the anterior lobes (left side). Scale bar, 100 mm.

(e) Cell cycle analysis of freshly isolated GNPs from P7 WT and *Chd7* homozygous mutant [*Atoh1-Cre::Chd7$^{f/f}$*] pups injected with BrdU and sacrificed 2 hour later. Note that there is no significant change of the percentage of cells in individual cell cycle phases between WT and Chd7 mutant cells.

(f) Immunostaining of BrdU in cerebella from P7 Chd7 WT and [*Atoh1-Cre::Chd7$^{f/f}$*] mutant mice administered with BrdU at P5. Sections were counterstained with Hematoxylin. Quantification of the number of BrdU-positive cells in IGL (middle panel) and the percentage of BrdU-positive cells in EGL, ML and IGL (right panel) is shown. Bars represent the mean value ± s.d. from three independent mice for each group. For middle panel, two-tailed t-test with equal variance was performed, p=0.0064. Scale bars, 100 mm. ML: molecular layer.

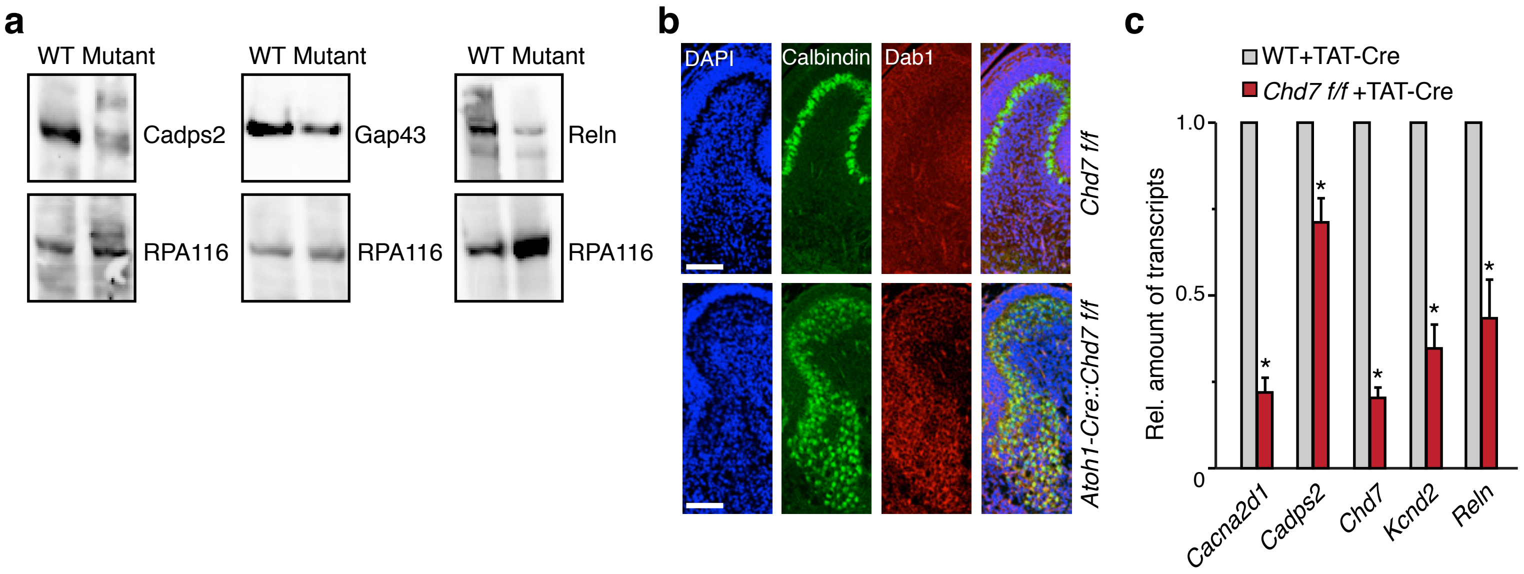

Supplementary Figure 4: Validation of Chd7 target genes.

(a) Western blot analysis of Cadps2, Gap43 and Reln in WT or *Chd7* homozygous mutant GNPs cultured without Shh for 48 hours. RPA116 was used as a loading control.

(b) Immunostaining of Calbindin and Dab1 in P3 WT or *Chd7* homozygous mutant [*Atoh1-Cre::Chd7*$^{f/f}$] cerebella. Note that the increased expression of Dab1 in mislocalized Purkinje cells in *Chd7* mutant cerebellum. Scale bars, 100 mm.

(c) qRT-PCR analysis of WT or *Chd7*$^{f/f}$ GNPs cultured without Shh and treated with 0.5 mM of TAT-Cre (Millipore) for 48 hours. Bars represent normalized mean value ± s.d. from 3 independent samples for each group. Paired two-tailed t-test with equal variance was performed. P = 0.00087 (for *Cacna2d1*); p = 0.019 (for *Cadps2*); p = 0.00053 (for *Chd7*); p = 0.0041 (for *Kcnd2*); p = 0.013 (for *Reln*).

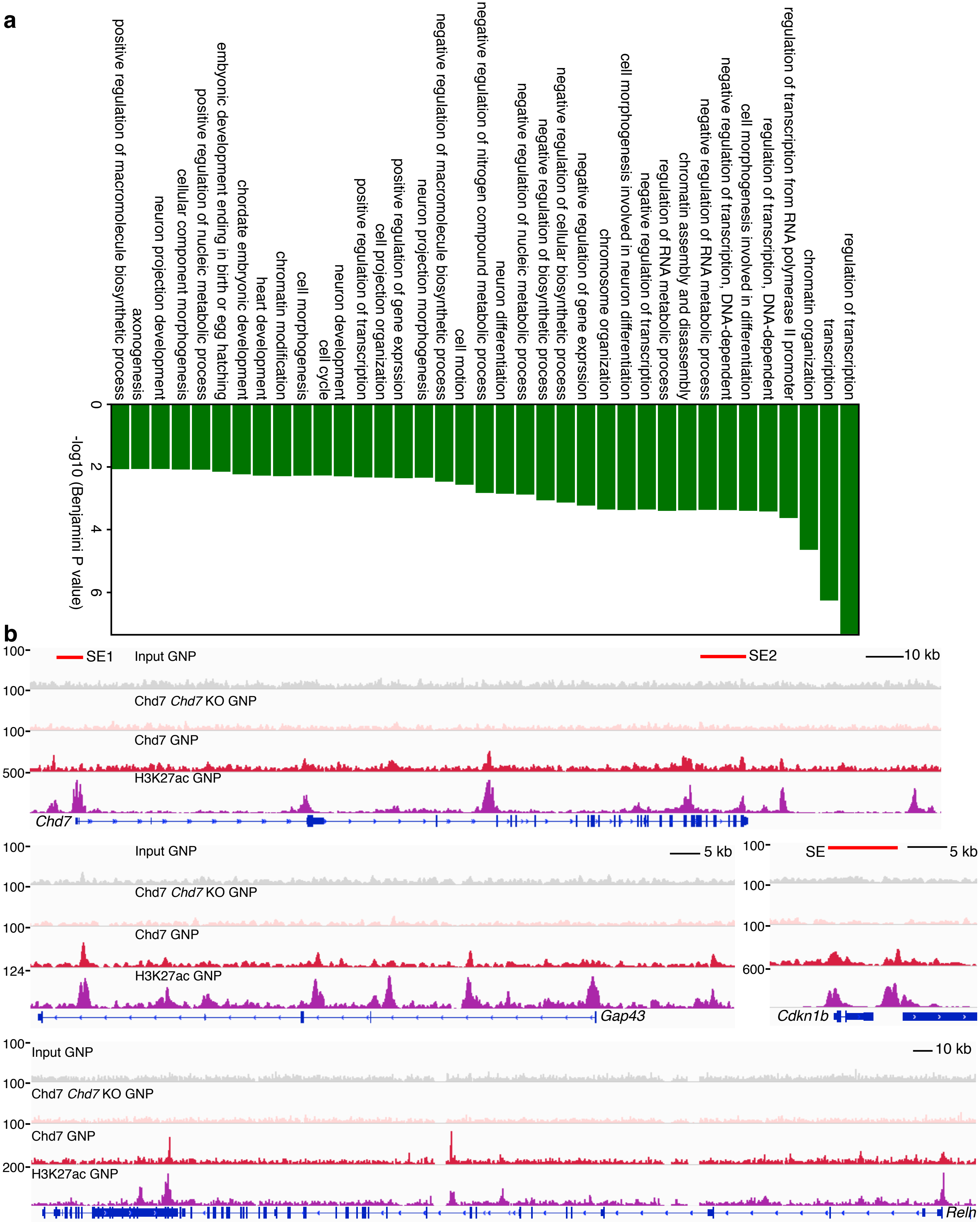

Supplementary Figure 5: Analysis of Chd7 ChIP-seq data.

(a) DAVID Gene Ontology Biological Process analysis of genes bound by Chd7 in GNPs identified by ChIP-seq. GO terms with Benjamini adjusted p-value <0.01 are shown.

(b) IGV track view of ChIP-seq density profile for Chd7 and H3K27ac in represented regions. Super enhancer regions (SE) are indicated by red lines.

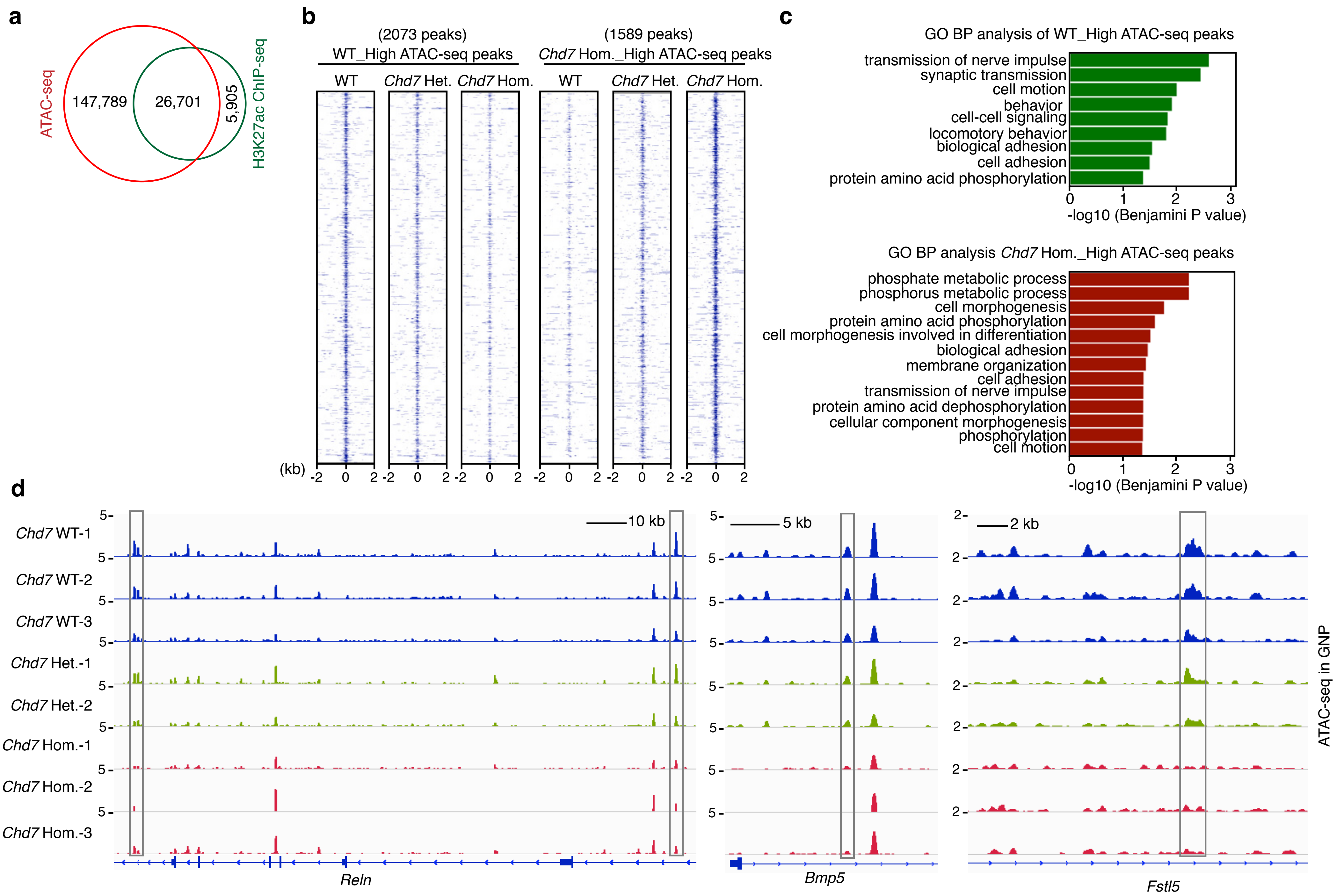

Supplementary Figure 6: Analysis of ATAC-seq data.

(a) Venn diagram shows the majority (81.9%) of H3K27ac ChIP-seq peaks overlapping with ATAC-seq peaks.

(b) ATAC-seq density heatmaps depict altered peaks (p<0.01, fold change>2) between WT and *Chd7* mutant GNPs. Regions within +/-2 kb of the ATAC peak center are shown.

(c) DAVID GO BP analysis of genes with altered ATAC-seq peaks between WT and *Chd7* mutant [*Atoh1-Cre::Chd7*$^{f/f}$] GNPs. GO terms with Benjamini adjusted p-value <0.05 are shown.

(d) Track view of ATAC-seq density profile (normalized to per million reads for each sample) at selected regions. The decreased ATAC-seq peaks are highlighted with gray rectangles.

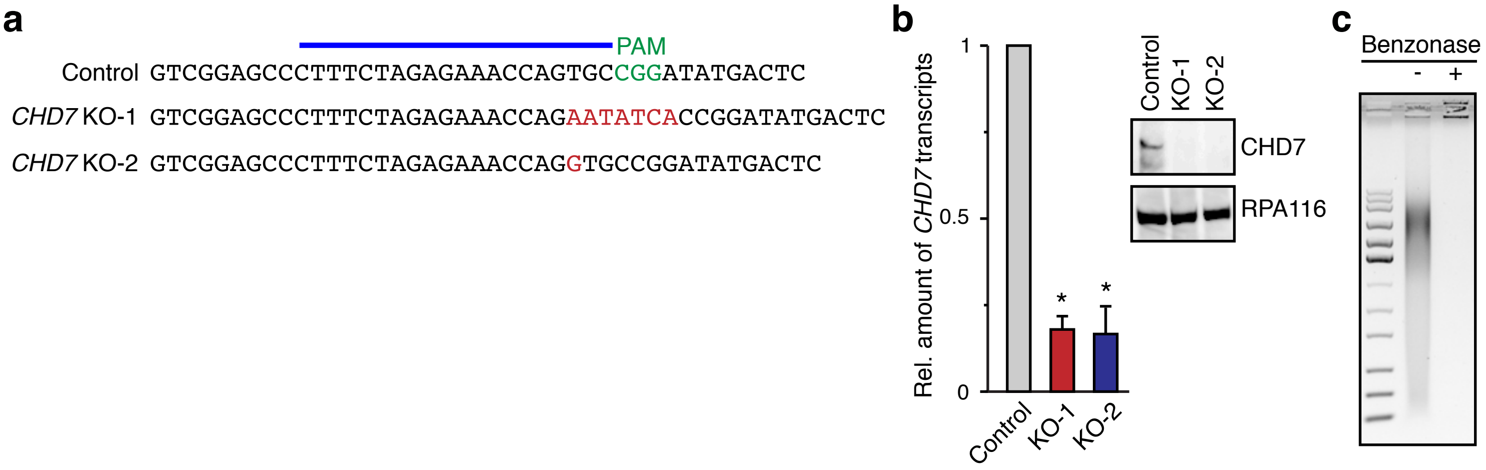

Supplementary Figure 7: Generation of CHD7-KO HEK293T cells.

(a) Genomic region of the *CHD7* gene targeted by sgRNA for CRISPR/Cas9-mediated mutagenesis. The DNA sequence recognized by the sgRNA is indicated by a blue line. The PAM motif is shown in green. The indels found in two independent *CHD7* KO cell clones are shown in red. PAM, protospacer adjacent motif.

(b) qRT-PCR (left panel) and Western blot (right panels) showing the depletion of CHD7 in *CHD7* KO cells. The level of GAPDH transcripts was used for the normalization of qPCR. RPA116 was used as a loading control for WB. Bars represent normalized mean value ± s.d. from 2 samples from each group. Paired two-tailed t-test with equal variance was performed, $p = 0.022$ (for KO-1); $p = 0.045$ (for KO-2).

(c) Agarose gel image shows complete digestion of DNA upon Benzonase treatment of nuclear extracts used for immunoprecipitation assays.

a

	GNPs	CGNs	p
Top1	2202	1849	0.0096
Top2a	5736	645	2.42E-05
Top2b	2673	3024	0.085

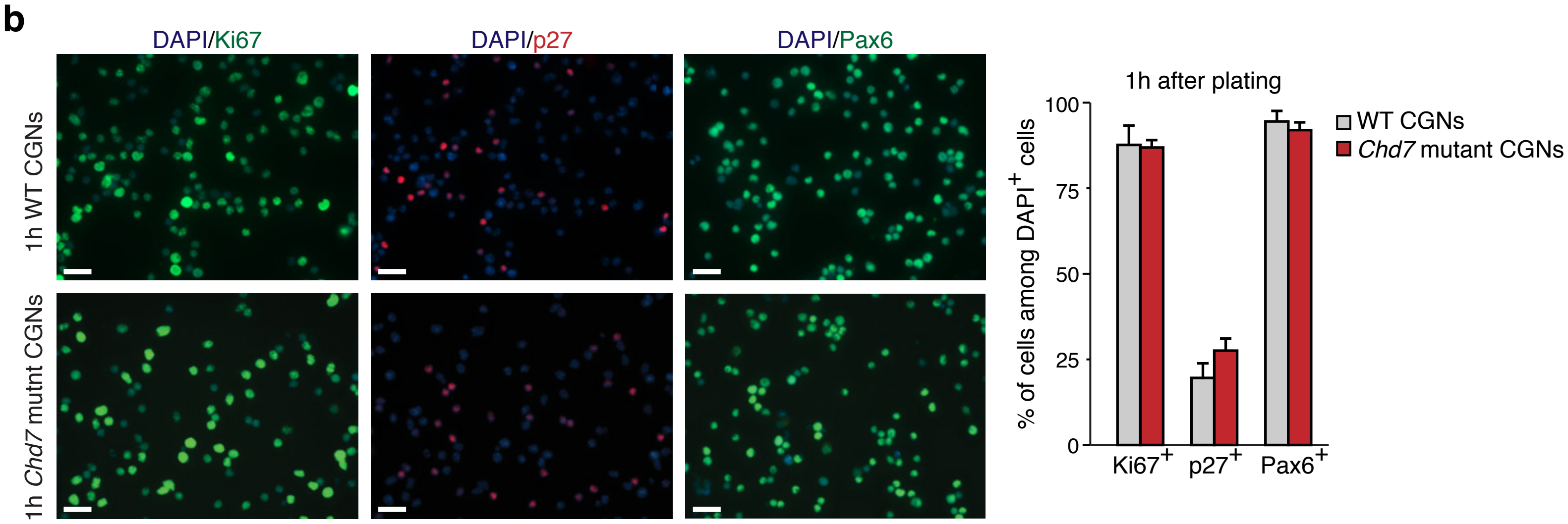

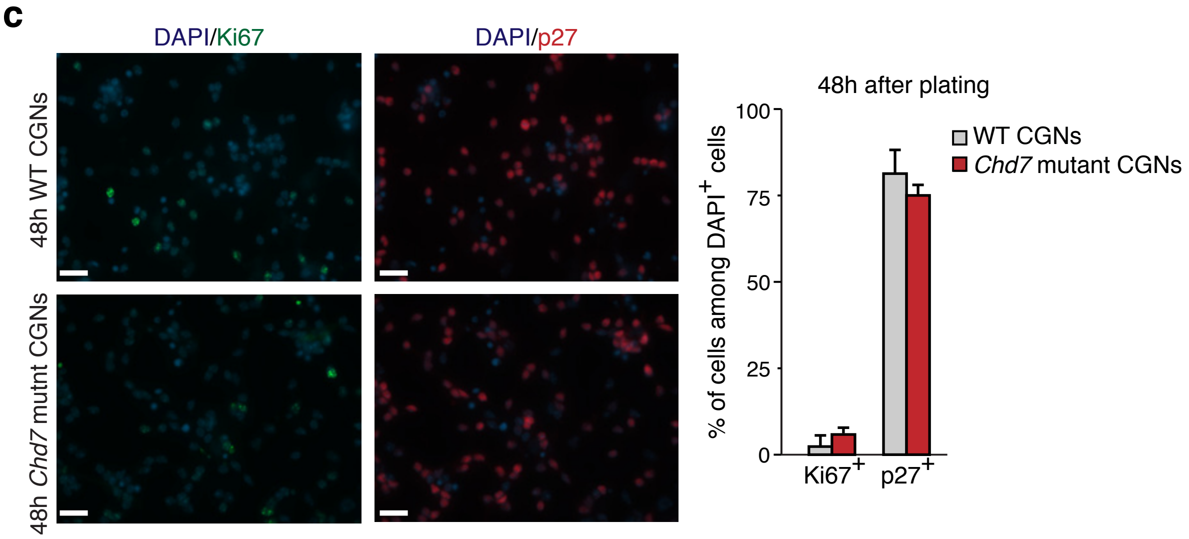

Supplementary Figure 8: Quantitative analysis of WT and *Chd7* mutant CGNs.

(a) The average expression value of *Top1*, *Top2a* and *Top2b* analyzed by microarray in granule cells treated with SAG (proliferating GNPs) or without SAG (postmitotic CGNs) for 48 hours. Value is the average number from 3 samples for each group. p value is shown.

(b) Immunostaining analysis of a proliferating marker Ki67, a differentiation marker p27 and a cerebellar granule cell marker Pax6 on isolated GNPs 1 hour after plating in culture medium without Shh. Quantification of the percentage of labeled cells is shown on the right panel. Scale bars, 20 mm.

(c) Immunostaining of Ki67 and p27 using isolated GNPs, after plating in culture medium without Shh for 48 hours. Quantification of the percentage of cells is shown on the right panel. Scale bars, 20 mm. For all quantification, five random selected area of each of three samples from each group were counted.

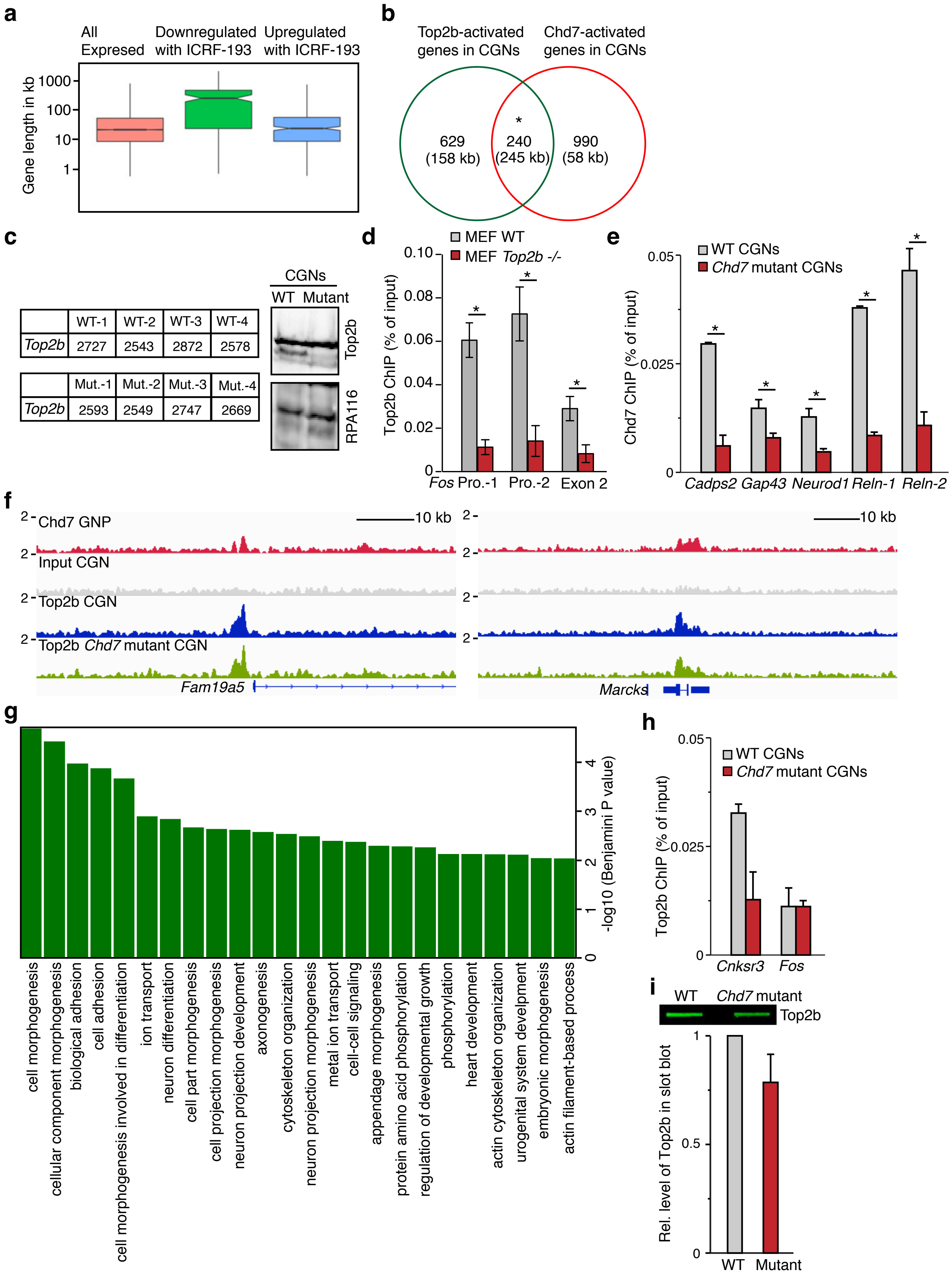
a
All Expresed
Downregulated with ICRF-193
Upregulated with ICRF-193
Gene length in kb
1000
100
10
1
b
Top2b-activated genes in CGNs
Chd7-activated genes in CGNs
629 (158 kb)
*
240 (245 kb)
990 (58 kb)
c
WT-1 WT-2 WT-3 WT-4
Top2b 2727 2543 2872 2578
Mut.-1 Mut.-2 Mut.-3 Mut.-4
Top2b 2593 2549 2747 2669
CGNs
WT Mutant
Top2b
RPA116
d
MEF WT
MEF Top2b -/-
Top2b ChIP (% of input)
0.10
0.08
0.06
0.04
0.02
0
Fos Pro.-1
Pro.-2
Exon 2
e
WT CGNs
Chd7 mutant CGNs
Chd7 ChIP (% of input)
0.05
0.025
0
Cadps2
Gap43
Neurod1
Reln-1
Reln-2
f
Chd7 GNP
Input CGN
Top2b CGN
Top2b Chd7 mutant CGN
10 kb
Fam19a5
Marcks
g
-log10 (Benjamini P value)
cell morphogenesis
cellular component morphogenesis
biological adhesion
cell adhesion
cell morphogenesis involved in differentiation
ion transport
neuron differentiation
cell part morphogenesis
cell projection morphogenesis
neuron projection development
axonogenesis
cytoskeleton organization
neuron projection morphogenesis
metal ion transport
cell-cell signaling
appendage morphogenesis
protein amino acid phosphorylation
regulation of developmental growth
phosphorylation
heart development
actin cytoskeleton organization
urogenital system develpment
embryonic morphogenesis
actin filament-based process
h
WT CGNs
Chd7 mutant CGNs
Top2b ChIP (% of input)
0.05
0.025
0
Cnksr3
Fos
i
WT
Chd7 mutant
Top2b
Rel. level of Top2b in slot blot
1
0.5
0
WT
Mutant

Supplementary Figure 9: Chd7 interacts with Top2b to activate a common set of neuronal genes in CGNs.

(**a**) Graph shows the distribution of gene length of all active genes in CGNs, significantly downregulated or upregulated genes ($p > 0.05$, fold change > 1.5) in CGNs upon treatment of ICRF-193, as compared to DMSO-treated cells.

(**b**) Venn diagram shows the number and average gene length of overlapped significantly downregulated genes ($p < 0.05$) in *Chd7* homozygous mutant and ICRF-193 treated CGNs, as compared to WT or DMSO-treated CGNs, respectively. $p < 2.2E-16$ (Chi-squared test).

(**c**) The expression value of *Top2b* of each WT and *Chd7* homozygous mutant CGNs in microarray analysis (left panel). Western blot displays the expression level of Top2b protein in WT and *Chd7* mutant CGN. The level of RPA116 is used as loading control.

(**d**) Chromatin immunoprecipitation assay shows the specificity of Top2b antibody used in this study. The binding of Top2b to the promoter and exon regions of the *Fos* gene in WT and *Top2b -/-* MEF is shown. Bars represent mean value ± s.d. from three independent experiments. Two-tailed *t*-test with equal variance was performed, $p = 0.0048$ (for Pro.-1); $p = 0.015$ (for Pro.-2); $p = 0.041$ (for Exon-2).

(**e**) ChIP assays using Chd7 antibody in WT and *Chd7* homozygous mutant CGNs. The PCR amplified regions were selected according to the Chd7 ChIP-seq peaks in GNPs. Bars represent mean value ± s.d. from two independent experiments. Two-tailed *t*-test with equal variance was performed, $p = 0.0051$(for *Cadps2*); $p = 0.048$ (for *Gap43*); $p = 0.033$ (for *Neurod1*); $p = 0.00045$ (for *Reln-1*, intron 10); $p = 0.015$ (for *Reln-2*, intron 43).

(**f**) Track view of ChIP-seq density profile for Chd7 in GNP and Top2b in WT and *Chd7* homozygous mutant CGNs in represented regions.

(**g**) DAVID GO Biological Process analysis of genes with significantly decreased Top2b ChIP-seq peaks ($p < 0.01$, fold change > 2) in *Chd7* mutant CGNs as compared to WT cells. GO terms with Benjamini adjusted p-value <0.01 are shown.

(**h**) ChIP assays using Top2b antibody in WT and Chd7 mutant CGNs. Bars represent mean value ± s.d. from two independent experiments. Two-tailed *t*-test with equal variance, $p = 0.0545$ (for *Cnksr3*); $p = 1$ (for *Fos*).

(**i**) The amount of Top2b covalent complexes in *Chd7* WT and mutant CGNs. The top panel shows a represented dot blot of Top2b. The quantification data is shown in the lower panel. Bars represent normalized mean value ± s.d. from 3 independent samples from each group. Paired two-tailed *t*-test with equal variance was performed, $p = 0.2169$.

Figure 3e

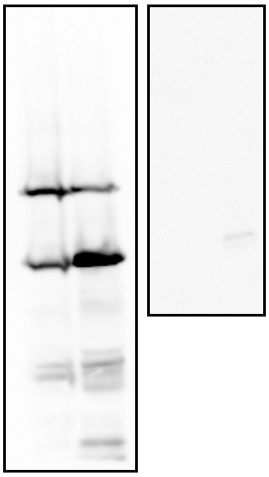

Figure 5b

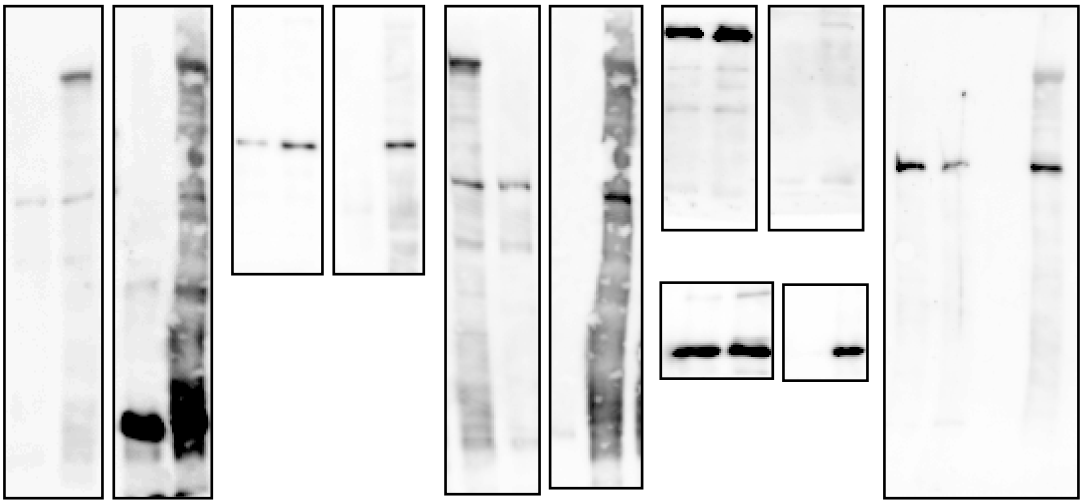

Figure 5c

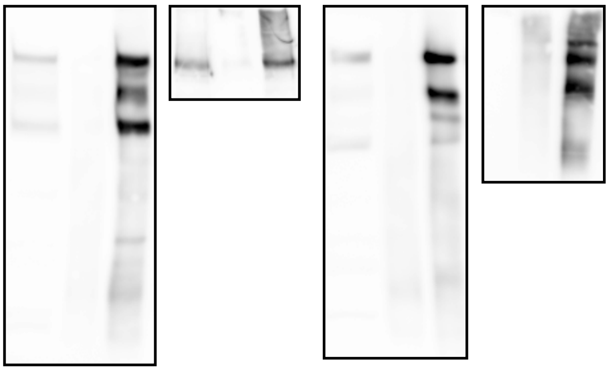

Figure 5d

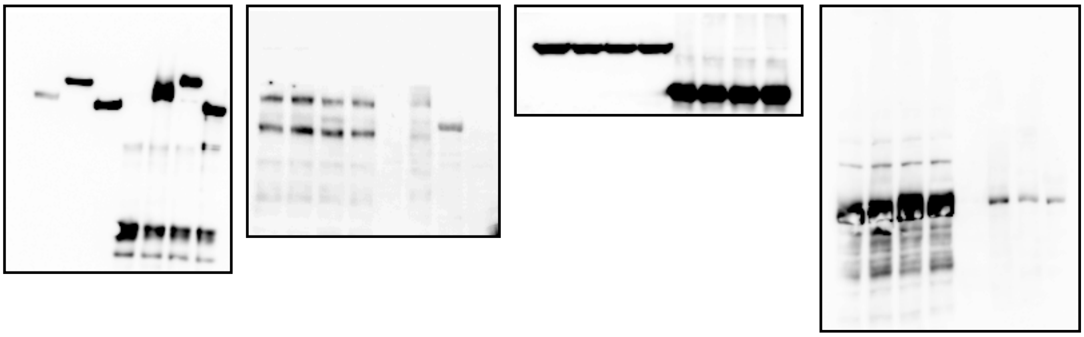

Supplementary Figure 4a

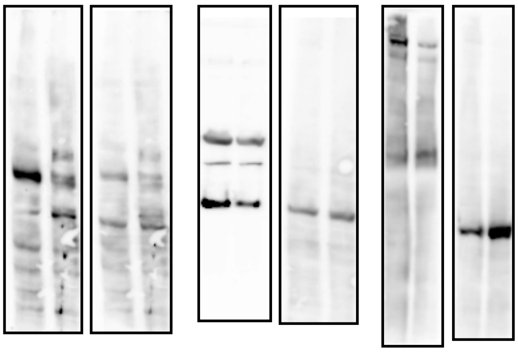

Supplementary Figure 7b

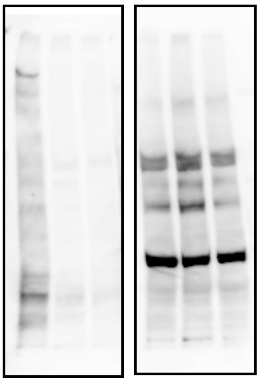

Supplementary Figure 9c

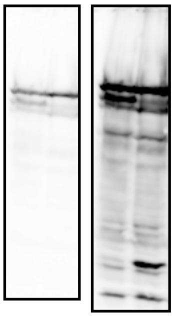

Supplementary Figure 10: uncropped pictures of western blots presented in this study.

Supplementary Table 1

The number of Chd7-bound genes identified by Chd7 ChIP-seq in RNA-seq and ATAC-seq analyses using *Chd7* WT and homozygous mutant GNPs.

	**RNA-seq down ↓ in *Chd7* KO**	**RNA-seq up ↑ in *Chd7* KO**	**Total**
**ATAC-seq peak up ↑**	18	10	28
**ATAC-seq peak down ↓**	21	6	27
**ATAC-seq peak both up ↑ and down ↓**	13	6	19
**Total**	52	22	74

Supplementary Table 2: Primers Used in the Study

Name	Sequences	Usage
Chd7 flox GT	Forward: TGCAGATGGGACGTTTTCAG<br>Reverse: CTGCAAGAACACAGGGCAAG	Genotyping
Chd7-GFP GT	Forward: CAGACAAGCTCCCAGGCTGTGTT<br>Reverse: TAGCGGCTGAAGCACTGCA	Genotyping
Cre GT	Forward: AGCGATCGCTGCCAGGAT<br>Reverse: ACCAGCGTTTTCGTTCTGCC	Genotyping
Chd7 flox recombination	Tm1C-Forward: AAGGCGCATAACGATACCAC<br>Floxed LR-Reverse: ACTGATGGCGAGCTCAGACC<br>Chd7-Rerverse: GCAAGAACACAGGGCAAGAA	Genotyping
ActB	Forward: CACCGGAGAATGGGAAGCCGA<br>Reverse: TCCACACAGATGGAGCGTCCA	Genotyping
Rosa26-CAG-LSL-Cas9-P2A-EGFP	Forward: CCCTCGTGATCTGCAACTCCAGTCTTTCTA<br>Rev.1: TAGGGGGCGTACTTGGCATATGATACACTT<br>Rev.2: CCCGACAAAACCGAAAATCTGTGGGAAGTC	Genotyping
Chd7 Taqman probe	Mm01219527_m1 ThermoFisher Scientific	qRT-PCR
Gapdh Taqman probe	Mm99999915_g1 ThermoFisher Scientific	qRT-PCR
CHD7 (human)	Forward: CAAAGCAGGGCCAGAACAAG<br>Reverse: TCCCACGTGCTGTCTTCATA	qRT-PCR
Cacna2d1	Forward: CAAGCGGAACAGACTTCTGATGGT<br>Reverse: AGTAGGTAGTGTCTGCTGCCAGAT	qRT-PCR
Cadps2	Forward: AAAGTGGAGGATGCTCTGCT<br>Reverse: TTCTCCCCTGTAAATGGCGT	qRT-PCR
Ccnd1	Forward: CCGGCCCGAGGAGCT<br>Reverse: ATGGCGGCCAGGTTCC	qRT-PCR
Gapdh	Forward: GTGTTCCTACCCCCAATGTGT<br>Reverse: ATTGTCATACCAGGAAATGAGCTT	qRT-PCR
Grik2	Forward: GATCGGGGAAGTGGGTGCCG<br>Reverse: GCTCCCATAGGGCCAGATTCCA	qRT-PCR
Kcnd2	Forward: CACAACAAGGAGTCACCAGCACTT<br>Reverse: GCTGTGGTCACGTAAGGTTGTTCA	qRT-PCR
Reln	Forward: CTGTGTCATACGCCACGAACA<br>Reverse: GGGGAGGTACAGGATGTGGAT	qRT-PCR
Cadps2 enhancer	Forward: GATAGCAGACCCCCAAATGA<br>Reverse: ATCTCAGGCCAGAGTGAACC	ChIP qPCR
Cnksr3 promoter	Forward: CACTCCAGTGCCCAACTCTT<br>Reverse: ACAGCGAAGCTGCTTACCAT	ChIP qPCR
Fos promoter-1	Forward: GAAAGCCTGGGGCGTAGAGT<br>Reverse: CCTCAGCTGGCGCCTTTAT	ChIP qPCR
Fos promoter-2	Forward: CATCTGCGTCAGCAGGTTTC<br>Reverse: GACTTCCTACGTCACTGGGC	ChIP qPCR
Fos exon-2	Forward: GCCAACTTTATCCCCACGGT<br>Reverse: TCTTCACCATTCCCGCTCTG	ChIP qPCR
Gap43 enhancer	Forward: GCCAAAGTGATGGGAAACAA<br>Reverse: TGTCCTTCCTCATTGGACCT	ChIP qPCR
Neurod1 promoter	Forward: TGAACAGGGAGAGAGGCAAG<br>Reverse: CCATTTTGCAGTGGACTCCT	ChIP qPCR
Reln intron 10	Forward: CTCTCTAGGGCCGGACTACC<br>Reverse: GCACCAGGCAGATTTCTTTC	ChIP qPCR
Reln intron 43	Forward: CATGGCCTTGAAAACACTCC<br>Reverse: TTTGTGCGAACACCACTAGG	ChIP qPCR
